# A Review Focused on the Psychological Effectiveness of Tai Chi on Different Populations

**DOI:** 10.1155/2012/678107

**Published:** 2011-07-18

**Authors:** Long Zhang, Charles Layne, Thomas Lowder, Jian Liu

**Affiliations:** ^1^Department Of Physical Education, Shenzhen Polytechnic, Shenzhen 518055, China; ^2^Department of Health And Human Performance, The University of Houston, Houston, TX 77204-6015, USA

## Abstract

As a popular exercise form, Tai Chi (TC) has been investigated to determine its contributions to an active and healthy lifestyle. There are an increasing number of researchers who focus on exploring the potential physiological and psychological benefits of TC but only a few systematic reviews of these benefits to a variety of populations. The purpose of this paper is to comprehensively evaluate the reported psychological benefits associated with practicing TC. Although many investigators have reported possible psychological benefits of TC for children, young adults, older healthy adults, and for a variety of patient populations, many of the reports suffer one or more methodological flaws. These flaws include inadequate study design, including lack of control groups, small sample sizes, unsophisticated statistical techniques, or publication without rigorous peer review. After reviewing the results of the existing literature regarding the potential psychological benefits of TC, we recommend that future investigations be conducted with additional adherence to the traditional scientific process.

## 1. Introduction

Increasing amounts of scientific evidence suggest that TC is related to improvements in mental health, psychosocial well-being, stress reduction, and self-reported sleep duration [1–5]. These benefits have been reported across a range of populations including children, healthy young and old adults and patient population such as those individuals with Parkinson's, cardiovascular disease, and AIDS. Other investigators have cast doubt on these findings by reporting little or no psychological benefits resulting from practicing TC. The objective of this paper is to summarize the existing literature concerning the potential psychological benefits of TC, including the physiological processes reported to be related to psychological variables, across a variety of participant groups. Additionally, recommendations are provided concerning the conduct of future investigations involving TC.

## 2. The Mechanism That Physiology Responds to Exercise to Produce Psychological Benefits

As a form of exercise, there is every reason to assume that the body's physiological systems respond to TC training in the same manner these systems respond to other forms of moderately intense exercising. This assumption includes those responses that have a directly mediating effect on psychological variables. Evidence that physiological factors may explain the beneficial effect of exercise on psychological well-being derives from research showing that exercise promotes the secretion of serotonin and dopamine, which in turn enhances aminergic synaptic transmission in the central nervous system [6]. Evidence from animal studies suggests that exercise stimulates the secretion of endogenous opioids (endorphins) and produces a state of euphoria [7]. Shyu Bai-Chuang et al. [8] also suggest that long-lasting muscle exercise (e.g., jogging) in rats produces discharges in muscle afferents which influence central endorphin mechanisms giving analgetic effects, and as a result mediated increase in pain threshold of the exercising rats. John Rodgers and Jill Randall (1985) report their study on “social conflict” analgesia in intruder mice which follows attack from aggressive residents; their investigation shows how opioid antagonists and cross-tolerance with morphine can be used to establish opioid involvement in an originally nonopioid, that is, behaviourally induced, form of analgesia. The reader is referred to Steinberg Hannan and Elizabeth Sykes [9].

### 2.1. Exercise and Human Psychophysiological Responses

Regular exercise is effective in preventing or ameliorating the metabolic and psychological comorbidities induced by chronic stress. These benefits are attributed to a main effect of exercise in reducing the sensitivity to stress and also subsidiary actions influencing metabolic functions and, especially, insulin sensitivity and the partitioning of fuels toward oxidation rather than storage [10]. 

According to a physiological mechanism, Jin [11] illustrated that TC training could increase noradrenaline excretion in urine, decrease salivary cortisol levels, and raise heart rate, leading to a less general mood disturbance with feelings of more vigour and less tension, anger, fatigue, confusion, state anxiety, and depression. A following study suggested that, whilst enhancing vigour, Tai Chi could reduce mental and emotional stress, reduce salivary cortisol levels, and improve mood states, although the author admitted that this could be due to the high expectancy level of people practicing Tai Chi [12]. A study of adolescent females with mild depression suggested that regular exercise alleviates the depressive state and reduces the excretion of stress hormones for example urine cortisol and epinephrine [13]. Exercise also reduces oxidative stress and improves neuroendocrine autoregulation, which can counter stress-related neuronal degeneration [14]. TC exercise has been associated with significantly decreased sympathetic nervous system activity [15]. TC practitioners' psychological tensions may be alleviated by production of the regulatory T cell mediators transforming growth factor beta and interleukin 10 under specific antigen stimulation, which may improve mucosal defence leading to a lower risk of autoimmune and inflammatory disorders in adults [16, 17]. With regard to the underlying physiological effect of TC, autoregulatory signaling pathways may play a significant role; such pathways are represented in the brain by limbic reward and motivation circuitries [18].

These findings suggest that regular exercise training has beneficial antidepressant and anxiolytic effects, and these are associated with a decrease in the neuroendocrine response to stress.

Physical exercise increases brain-derived neurotrophic factor (BDNF) levels in the hippocampus, which can play an important protective role against hippocampal degeneration which is associated with chronic stress [19]. The hippocampus is a target of stress hormones and can be particularly vulnerable to neuronal degeneration [20]. Increasing evidence suggests that moderate exercise could increase proliferation of hippocampal neurons and provide a balancing force against age-related neuronal loss, consequently slowing the decline in hippocampal volume. Davidson et al. [21] proposed an active role of the hippocampus in emotional responding. They hypothesized that individuals habitually failing to regulate their affective responses in a context-sensitive fashion might have a functional impairment of the hippocampus. Thus, larger hippocampal volumes may account for exercise practitioners' singular abilities and habits to cultivate positive emotions, retain emotional stability, and engage in mindful behavior. 

An anterior excise study was performed by The National Aeronautics and Space Administration-United States Public Health Service Health Evaluation and Enhancement Program (NASA-USPHS) [22]. They found that improvement in physical fitness level was related positively to the subjective reports of less strain and tension, better health, and promoted stamina as well as more positive work attitudes. A latest review focused on examining the effect of exercise training on anxiety symptoms among patients, following scrutinizing forty English-language articles in scholarly journals involving sedentary adults with a chronic illness, calculating the Hedges *d *effect sizes from studies of 2914 patients and extracting information regarding potential moderator variables, and adopting random effects models to estimate sampling error and population variance for all analyses scientifically concludes that exercise training reduces anxiety symptoms among sedentary patients who have a chronic illness. The study results also provide clinicians with solid evidence to recommend exercise training to patients as a means for reducing anxiety symptoms with minimal risk of adverse events [23].

In conclusion, exercise improves physiological function of human being, including metabolic level, neurons proliferation, and neuroendocrine autoregulation, which benefit psychological performance directly or indirectly, such as positive emotion, emotional stability, stress tolerance, and anxious relief.

## 3. Mind-Body Movements from China

### 3.1. TC

In the past, the term TC has been used in connection with philosophy, religion, literature, art, medicine, and even astronomy [24]. The modern use of TC refers to a standardized series of movements practice by approximately two hundreds and fifty millions people worldwide [25]. While undoubtedly practiced prior to the earliest written references, TC has been reported to have been invented by a Taoist priest named Chang San Feng in the 15th Century [26]. Other historical references credit Wangting Chen a martial art master from the Chen village for inventing TC in the mid-1600s [27–29]. As a branch of martial arts, TC has been practiced for much longer than 400 years and its origin cannot be attributed to a single individual. TC has evolved into its current form that combines distinct and identifiable movements designed to promote physical and psychological well-being. As such, TC is becoming a more accepted method of exercise to promote a healthy lifestyle. In the West, TC is classified in the category of alternative medicine. Naturally, as TC gains expanded acceptance in the West, it has become the focus of increased attention by scientific investigator and health care professionals.

### 3.2. Qigong

Qigong and TC are the two most popular Chinese medical exercises which have been practiced for many hundreds of years. The two movement forms combine gentle, mind-body, and therapeutic movements and are therefore suitable for people of different ages with various health conditions. There is growing evidence to suggest that these exercises have many health benefits and as a result are becoming increasingly popular in the West. Based on the data obtained from National Health Interview Survey (NHIS), Barnes reported that in the United States alone an estimated 2,500,000 and 500,000 individuals use TC or qigong, to improved their overall health status [30].

Qigong is closely related to TC and is also a historical derivative of Chinese healing practices. Like TC, it is believed to manipulate Qi (vital energy) through mind-body exercises [31]. Generally speaking, Qigong is a meditative form of exercise that, like TC, requires coordinated, gentle movements with mental focus, breathing, and relaxation for physical, mental, and/or spiritual cultivation [32]. The primary difference between Qigong and TC is that Qigong manipulates Qi by mindfulness more than by movements, the opposite being true for TC.

## 4. Psychological Benefits of TC

As a form of exercise built upon the mind-body connection, TC combines physical movement, meditation, and breathing to induce relaxation and tranquility of the mind and improves balance, postural control, movement coordination, and muscle endurance, strength, and flexibility. It can be practiced by people of all ages, physical statures, strength, and physical abilities because it relies more on technique than strength.

TC as a mind-body movement program results in the integration of physiological and psychological benefits, including improved confidence, quality of life (QOL), motivation, self-efficacy, and overall mood elevation. Significant improvements have been detected for QOL dimensions such as general health perception, social functioning, vitality, and mental health or psychological well-being [33–35]. Kin et al. [36] used the subscores for physical functioning (PF), physical role (PR), bodily pain (BP), general health (GH), vitality (VT), social functioning (SF), role emotional (RE), and mental health (MH) as assessed by the SF-36, to evaluate the HLQOL (the health-related quality of life) of TC practitioners with those obtained from age-matched national standards for correlative age groups. The authors found that in the 60- to 69-year-old TC practitioners had significantly higher PF, GH, and MH than the national averages and the 70- to 80-year-old subjects in this study had significantly higher PF, PR, BP, GH, VT, RE, and MH than the national averages; additionally the duration and the frequency of practicing Tai Chi statistically significantly correlated with MH and PF, and GH and PF, respectively. Other authors have reported that participating in TC led to significant improvements in body care and health behaviors due to improved motivation [37, 38]. Significant psychosocial benefits have also been observed, such as better perceived social support, mood state, confidence, self-efficacy, and superior relaxation among TC practitioners [4, 39–41]. 

TC practice is also associated with significant decreases in sadness, confusion, anger, tension, and fear as well as with increases in energy and happiness [42], self-esteem [43], and self-efficacy [44]. Some practitioners of TC reported improved quality of sleep due to combined TC and mindfulness-based stress reduction [45]. Furthermore, in a randomized controlled trial, TC was more effective than neutral reading in reducing state of anxiety in TC practitioners; additionally the stress-reduction effect of TC was similar to moderate physical exercise [12]. 

In summary, the literature supports the idea that practicing TC has a multitude of positive psychological benefits that promote overall psychological well-being including the reduction of mental health disorders, anxiety, depression, or phobias [46].

## 5. TC's Psychological Benefits for Different Populations

### 5.1. Children and Teens

A randomized controlled trial conducted by Baron [47] examined the psychological effect of a 12-week (1 h, 2 times/wk) TC program on 90 school children, grades 4 to 6 (52 boys, 38 girls), who were pretested on psychological measures including those of perceived self-competence, visual-motor integration, and anxiety. The author reported that the TC group significantly improved their scores on the perceived self-competence and visual motor integration tests. However, the results of this study must be interpreted cautiously as no age-matched control group was included. Given the age of the study's population, an age when children are undergoing rapid physical and psychological changes, it is difficult to attribute the reported benefits exclusively to the practice of TC. A nonrandomized controlled study of 22 young people (mean age 21 years) found that 20 days of TC practice significantly decreased nightmares [48]. Baron and Faubert [49] reported the findings of a single-case research design which examined the effects of 10-week TC sessions (1 hour per session, two sessions per week) on state anxiety and mood of children with severe learning disabilities.The participants were three upper elementary children (mean age is equal to 13.3 years). The State-Trait Anxiety Inventory for Children (STAIC), a-state scale, a 28-item mood inventory, and Conners' Teacher Rating Scale-39 (CTRS-39), a scale used to assess whether a child has attention deficits and/or hyperactivity, were completed prior to, during, and after the TC intervention. Results suggested that the practice of TC had the strongest effect on the child who presented with hyperactivity and heightened anxiety. The literature supports the psychological benefits of TC exercise on young teenagers as if it may be both a noninvasive and cost-effective alternative to the pharmacological therapies presently being prescribed in increasing numbers.

Wall et al. [50] conducted a pilot project with middle school students from an urban environment characterized by abuse of power and high level of exposure to aggressive behaviors. TC and MBSR (mindfulness-based stress reduction) programs were employed to determine if these interventions could improve a variety of psychological factors in these teenagers. Self-reports indicated that the participants experienced improved well-being, calmness, relaxation, sleep patterns, less reactivity, increased self-care, self-awareness, and a sense of interconnection with or interdependence on nature; moreover, it appeared that combined TC and MBSR could trigger interest of middle school-aged children. The results of this project implied that TC and MBSR might serve as a transformational tool for middle school-aged children and should be recommended for health promotion. No control group was included in this study.

### 5.2. Young Healthy Adults

A three-month intervention of TC exercise was conducted with 30 healthy college students enrolled in a university class. TC was practiced twice a week for one hour over the course of three months. Multidimensional physical (PHD) and mental health (MHD) scores were evaluated with the SF-36v2 health survey questionnaire before and after the intervention. The outcome showed that three of the four mental health variables significantly improved after the TC intervention. Improvement was reported in vitality (representing a sense of energy and freedom from fatigue), role mental/emotion function (representing the limitations in usual home or work activities because of emotional problems), and mental health. Additionally, the overall mental health dimension, which is a combination of the four mental health variables, also significantly improved. These results suggest that a TC program can improve the physical and mental health of college students [51]. 

Caldwell et al. [52] explored the possible benefits of a semester-long program of either Pilates or TC training for perceived self-efficacy, sleep quality, mood, strength and balance in college students. Subjects who served as the intervention groups were randomly selected from five physical education classes (three Pilates mat classes and two Tai Chi classes) and subjects who served as a control group were recruited from either an outdoor leadership or special recreational class. The TC classes were attended twice a week for 50 min each session for 15 weeks, during the first week of class, at the mid-term, and at the end of the semester. All subjects were asked to complete a survey instrument containing self-regulatory efficacy scales, sleep quality indexes, mood scales, and demographic questions during the initial week of class, mid-term, and again during the last week of class. Self-efficacy was found to be improved in the TC and Pilates groups, and sleep quality and mood were found trending towards improvement in the TC group as well; in the control group, self-efficacy was also found trending towards improvement, but sleep quality or mood was not found to be or trending to be improved. Therefore, the authors concluded that Pilates and TC were effective exercise modes to improve mental parameters in college-aged individuals.

Overall, current evidence suggests that TC is an effective exercise modality for improving the psychological well-being and mental health of young adults.

### 5.3. Healthy Adults

Brown et al. [39] evaluated the psychological changes associated with 16-week moderate- and low-intensity exercise training programs, two of which possessed a cognitive component. The study included 135 healthy, sedentary subjects (69 women, mean age 54.8 ± 8.3 yr, and 66 men, mean age = 50.6 ± 8.0 yr). All subjects were randomly assigned to a control group (C), moderate-intensity walking group (MW), low-intensity walking group (LW), low-intensity walking plus relaxation response group (LWR), or TC practice-mindful exercise group (ME). The results indicated that women in the ME group experienced reductions in tension, depression, anger, confusion, total mood disturbance, and an associated overall improvement in general mood. The authors concluded that exercise plus cognitive strategy training programs were somewhat more effective than exercise programs lacking cognitive component in promoting psychological benefits.

Esch et al. [18] conduced an eighteen-week study to investigate the subjective and objective clinical effects of TC intervention in 21 young healthy adults (27.14 ± 4.25). The subjects attended a 90-minute TC exercise session 12 times over 14 weeks (the left 4 weeks without formal guidance, as a follow-up stage for measures), conducted by an experienced and certified TC teacher from the Yongnian Taijiquan Association of Europe. The subjects' pre- to postphysiological (blood pressure, heart rate, and saliva cortisol) and psychological (SF-36, perceived stress, and significant events) parameters were measured and compared. Even though only nine participants completed all measurements, the summarized mental health measures all improved, strongly suggesting a positive benefit resulting from the practice of TC. Jin [11, 12] reported that the practice of TC can result in less general mood disturbance with feelings of increased vigour and decreased tension, anger, fatigue, confusion, state anxiety, and depression. Moreover, it has been reported that group programmes such as TC may counter social isolation and support positive mental health status [53]. In addition, practicing TC in a group settings can encourage the development of social support by practitioners forming friendships outside of the class and a sharing of similar problems [54]. 

Therefore, the increasing evidence supports that TC may be an appropriate candidate for integrative stress management options and related research for health adults.

### 5.4. Elderly

A number of authors have reported improved measures of psychological variables in elderly individuals who participate in TC. It has been reported that, in comparison with the low impact exercise control group, TC practicing group can achieve the increase of nighttime sleep duration combined with the decrease of daytime drowsiness by a 24-week TC practice program in elderly individuals [5].

Li et al. [2, 3] examined the effectiveness of a TC exercise program on enhancing elderly individuals' psychological well-being. A control group continued their daily routine activities while an intervention group participated in a TC class. Psychological well-being measures were assessed at baseline, three months, and after six months of Tai Chi training. Using repeated measures data analysis by latent growth curve modeling methodology, the results indicated that individuals who participated in the TC program showed higher levels of health perceptions, life satisfaction, positive affect, and psychological well-being. Additionally, lower levels of depression, negative affect, and psychological distress were also reported. This longitudinal study suggests that TC may be an effective form of exercise for improving psychological well-being in older adults.

A cross-sectional study aimed to investigate the effects of TC on the health-related quality of life (HRQOL) in the senior population indicated that TC improves quality of life among a random sample of elderly individuals living in Taiwan. In this study, 140 seniors (77 males and 63 females, aged 40–70 years) who regularly practiced TC served as subjects. The SF-36 questionnaire, which used 8 domains to evaluate the subjects' HRQOL, was administered. The resulting data were compared with those of 560 age- and sex-matched control subjects that were sampled from the general population (308 males and 252 females). Multiple regression analysis was used to compare the quality of life in each of the eight domains between the two groups. The results that showed the TC group had significantly higher quality-of-life scores than the control group in each of the eight domains with the exception of the bodily pain scales. Using multiple linear regression adjusted for covariates, the TC group had significantly higher scores in physical functioning, physical roles, general health, vitality, and social-functioning scales than the control group. In most of the domains in both the TC group and the control group, quality of life became declined with increased age; however the vitality and social-functioning domain scores of the TC group showed an opposite trend, either improving or remaining unchanged despite increasing age [55].

Hill et al. [56] reported that a 6-month physical activity program of TC leads to significant improvements in both physical and psychological health outcomes, including a reduction in symptoms of depression in elderly subjects. Chou et al. [57] reported that a TC practicing group had decreased depressive symptoms compared to control group after a 12-week TC program with elderly subjects. The practice of TC has been shown to improve perception of task-special personal efficacy of older adults [2, 3]. Hartman et al. [58] also reported that a 12-week TC program could enhance self-efficacy, reduce tension and nervousness, and increase social support in older patients with osteoarthritis.

A randomized controlled trial randomly allocated 112 healthy older adults aged 59 to 86 years to a TC or a health education group during a 25-week intervention program. Sleep quality, as assessed by the Pittsburgh Sleep Quality Index (PSQI), improved significantly more in the TC group than in the health education group. This included the sleep parameters of rated sleep quality, habitual sleep efficiency, sleep duration, and sleep disturbances [59].

In conclusion, the existing evidence suggests that TC, as a simple, low-cost exercise modality, can attenuate the age decline in physical function and achieve successful antiaging [60]; moreover, TC practice can also effectively improve psychological well-being in older adults, which may in turn improve overall quality of life.

### 5.5. Patients with Cardiovascular Diseases

In addition to providing psychological benefits to healthy individuals, TC has been reported to improve a number of psychological measures in a variety of patient populations. A 12-week TC program for those with borderline hypertension exhibited significant improvement in trait anxiety status relative to a sedentary control group. The authors concluded that TC might be an excellent alternative to drug therapy for mildly hypertensive patients [61].

Yeh et al. [62] randomly assigned thirty patients with stable chronic heart failure to receive either standard care (*n* = 15), which included pharmacologic therapy and dietary and exercise counseling, or 12 weeks of TC training (*n* = 15) in addition to the standard care. The subjects who were on average 64 (±13) years old received a 1-hour TC class twice a week for 12 weeks. Patients in the TC group showed improved quality-of-life scores accessed with the Minnesota Living with Heart Failure Questionnaire compared with patients in the control group. The authors concluded that TC leads to benefits that enhance quality of life and functional capacity in patients with chronic heart failure who are already receiving standard medical therapy. Barrow et al. [63] also found similar increase in quality of life, including overall psychological profile, following either a 16-week TC intervention or standard care for patients with heart diseases. Taylor-Piliae [64] concluded that TC training provided both physiological and psychological, cost-effective benefits when used as an adjunct to cardiac rehabilitation exercise program.

Finding an easy-to-perform and appropriate activity that patients will continue to practice is critical to therapeutic success. Clinical trials have reported excellent compliance with TC interventions and suggest that TC may promote exercise self-efficacy [40, 65]. Additionally, exercise is a well-documented and effective approach for secondary prevention in patients with diagnosed cardiovascular diseases. However, existing studies have consistently documented that conventional cardiac rehabilitation programs are underutilized [66]. Therapies such as TC may offer patients additional options, whether as a complement to formal cardiac rehabilitation, as a component of maintenance therapy, or as an alternative to move “traditional” exercise modes such as walking or swimming, which may increase program compliance [67].

### 5.6. Patients with Parkinson Disease (PD)

TC is an exercise intervention modality supported by the National Parkinson Foundation of the United States and Parkinson Disease Society in Canada [68]. The effectiveness of TC in delaying the negative clinical consequences of PD has been verified repeatedly [68–70].

Cheon et al. [71] evaluated the effects of TC on Parkinson patients who were nonrandomly assigned to a TC intervention, a combined exercise program, or a sedentary control group. After the 8-week intervention, quality-of-life scores were higher for the TC and combined exercise program group compared to the sedentary controls. Another uncontrolled clinical trial addressed the effect of TC on PD [72]. These authors found that TC had positive effects on depression but no effects on the overall quality of life.

A 12-week, 45-minute per week TC exercise intervention program with 8 PD patients and 7 support partners revealed that both groups perceived psychological and social benefits resulting from practicing TC. The psychological benefits included “feel more relaxed,” “calming effect of doing TC,” and “energy level somewhat improved.”

The authors suggested that their program encouraged social participation and support partner involvement might have a positive influence on exercise persistence, as well as the health and well-being of both the PD patients and their support partners [73].

### 5.7. HIV Patients (AIDS Patients)

McCain et al. [74] evaluated TC as a complementary intervention in a randomized clinical trial which included an HIV-positive control group, assessing specific biopsychosocial effects in individuals living with various stages of AIDS. Dependent measures included psychoneuroimmunology-based outcomes in the domains of psychosocial functioning (perceived stress, coping strategies, perceived social support, and psychological distress), quality of life (including spiritual/existential well-being), and physical health (neuroendocrine mediators, immune status, and disease progression). A total of 59 participants completed TC training, and 56 individuals completed the waitlisted control condition. Using multivariate analysis of covariance, the pre- to postintervention comparisons indicated that; after the intervention, those in the TC group had higher existential well-being, had higher perceptions of social support in the realm of guidance, and more frequently used appraisal-focused coping strategies.

Robins et al. [17] conducted a randomized clinical trial to determine whether three short-term stress management interventions (TC training, spiritual growth groups, and cognitive-behavioral stress management) improved psychosocial functioning, quality of life, and neuroendocrine-immune function in persons with varying stages of AIDS disease. Additionally, qualitative interviews were conducted with a randomly selected subset of participants to explore their unique stress experiences. The results indicated that the TC intervention may account for clinically meaningful improvements in psychosocial functioning and may moderate the progression of AIDS disease through enhanced quality of life and coping mechanisms. 

 A randomized clinical trial conducted by McCain et al. [75] was designed to test effects of three 10-week stress management approaches, including cognitive-behavioral relaxation training (RLXN), focused TC training (TCHI), and spiritual growth groups (SPRT), compared to a wait-listed control group (CTRL) with no treatment, 4 groups contained 252 individuals with HIV. The results indicated that both the RLXN and TCHI groups were found using less emotion-focused coping than the CTRL group, with all treatment groups having augmented lymphocyte proliferative function, which accordingly enhanced immune function to some degree. The findings of this study suggested that immune function and quality of life may be enhanced with cognitive-behavioral stress management, TC, and spirituality-based interventions. Overall, the above studies suggest that practicing TC can have positive psychological benefits to those who understand they are living with a medical condition which has a relatively high probability of significantly reducing their life expectancy.

## 6. Recommendation

Despite the multitude of studies reporting positive psychological benefits of practicing TC, TC has not gained universal acceptance in the culture of Western medicine as an effective alternative to more traditional form of exercise. A factor in this resistance may be attributed, in part, to the lack of scientific rigor in the experimental design of many of the studies reporting the psychological benefits of TC. Therefore, it is recommended that future studies should be concerned with experimental design, execution, analysis, and results reported by those researchers conducting TC- related research. In particular, attention should be paid to the training of the participants, qualifications of exercise instructors, the criteria for the inclusion and exclusion of the subjects, the inclusion of control groups, and the statistics used to evaluate the possibility of a successful TC intervention.

## 7. Summary

This paper has summarized many of the studies that have included psychologically based dependent measures. The existing data indicates that the TC provides positive psychological benefits to its practitioners including children, teenagers, young adults, older adults, and those with a variety of chronic health conditions. These psychological benefits are reported to include reductions in mood disturbances, sadness, anger, and confusion, as well as improvements in sleep quality, mood states, happiness and overall psychological well-being. In conclusion, the literature suggests that TC is a valuable method by which to enhance or maintain a healthy state of psychological functioning for a variety of populations. However, future research utilizing rigorous scientific design principles is necessary to further document the positive psychological benefits of TC and to gain a greater understanding of the mechanisms underlying these benefits.
